# Three-Dimensional Printing of PVA Capsular Devices for Applications in Compounding Pharmacy: Effect of Design Parameters on Pharmaceutical Performance

**DOI:** 10.3390/pharmaceutics16081069

**Published:** 2024-08-15

**Authors:** Juan Francisco Peña, Ivana Cotabarren, Loreana Gallo

**Affiliations:** 1 Planta Piloto de Ingeniería Química, PLAPIQUI (UNS-CONICET), Camino La Carrindanga Km 7, Bahía Blanca 8000, Argentina; jfpena@plapiqui.edu.ar (J.F.P.); lgallo@plapiqui.edu.ar (L.G.); 2 Departamento de Ingeniería Química, Universidad Nacional del Sur (UNS), Av. Alem 1253, Bahía Blanca 8000, Argentina; 3 Departamento de Biología, Bioquímica y Farmacia, Universidad Nacional del Sur (UNS), San Juan 670, Bahía Blanca 8000, Argentina

**Keywords:** magistral compounding, 3D printing, fused deposition modeling, printing parameters, capsular devices, drug release modeling

## Abstract

The creation of products with personalized or innovative features in the pharmaceutical sector by using innovative technologies such as three-dimensional (3D) printing is particularly noteworthy, especially in the realm of compounding pharmacies. In this work, 3D printed capsule devices (CDs) with different wall thicknesses (0.2, 0.3, 0.4, 0.6, and 0.9 mm) and sizes were designed and successfully fabricated varying printing parameters such as extrusion temperature, printing speed, material flow percent, and nozzle diameter. The physicochemical, pharmaceutical, and biopharmaceutical performance of these CDs was evaluated with the aim of achieving an immediate drug release profile comparable to hard gelatin capsules (HGC) for use in magistral compounding. It was observed that the disintegration time of the CDs increased with wall thickness, which correlated with a slower drug release rate. CDs with configurations presenting 0.4 mm wall thickness and sizes comparable to HGC n° 0, 1, and 2 demonstrated satisfactory weight uniformity, short disintegration times, and immediate drug release, indicating their potential as effective devices in future compounding pharmacy applications. In addition, a modified Weibull-type model was proposed that incorporates wall thickness as a new variable in predicting dissolution profiles. This model improves the process of selecting a specific wall thickness to achieve the desired dissolution rate within a specified time frame.

## 1. Introduction

Magistral compounding is defined as any medicinal product prescribed in a medical prescription for an individualized patient, subsequently prepared, packaged, and labeled by a pharmacist in the laboratory of their pharmacy and dispensed therein [1]. Magistral compounding has been, for centuries, and until a few decades ago, the only alternative to properly produce medications in pharmacies. However, industrialization has led to a certain degree of depersonalization of medication. In this sense, magistral compounding takes on a special role by providing a personalized approach to pharmacological treatment, adapting the medication to the unique physio-pathological characteristics of each person, thereby reducing the risk of potential adverse reactions [2,3]. In addition, magistral compounding can address therapeutic formulation gaps as orphan formulations, which are crucial for certain patients (children and elderly people) and for pathologies without specific industrial medicine (i.e., rare diseases) [4]. It is worth noting that magistral formulation has evolved by incorporating new pharmaceutical forms and small-scale procedures, along with rigorous quality controls for raw materials (drugs and excipients) and final formulations, aiming to reduce associated risks [5]. However, it remains an artisanal process that takes time and does not allow us to produce modified drug delivery systems [6]. Hard-gelatin capsules (HGC) are one of the most used pharmaceutical forms in magistral compounding, where hand-filling of one capsule at a time is a common practice. This method implies the introduction of the inverted capsule body into a pile of drug powder until the capsule body is filled up and has the desired weight. Another method employed is a capsule-filling jig, also called a hand-filling capsule machine. As can be seen, hand-filling is an easy but slow procedure that is not scalable to faster modes of production [7]. In this sense, the magistral compounding together with three-dimensional (3D) printing represents a promising tool to promote the improvement in the personalization of medicines [8]. Regarding this, Fused Deposition Modeling (FDM), a type of 3D printing technology, allows the design and production of tailored pharmaceutical dosage forms by transforming a 3D digital model into a 3D physical object. To this end, the printer deposits a polymeric material (in the form of filament) layer-by-layer under the control of computer software [9,10]. Hence, FDM 3D printing enables the personalization of medicine to produce both immediate and modified drug-delivery systems. Moreover, this technology offers the possibility of automating the printing process by incorporating multiple heads, thus accelerating the production process. Consequently, FDM process automation, coupled with high printing precision, reduces the risks associated with magistral compounding [11,12,13]. These characteristics position FDM 3D printing as a relevant technology to enhance the development of personalized pharmaceutical dosage forms in magistral compounding [6]. It is important to note that regulatory aspects of introducing 3D printing in the compounding pharmacy remain a challenge. This will require validating 3D-printed medicine, including quality assurance for raw materials, software, design files, and printers [8]. For FDM 3D printing of pharmaceutical forms, achieving a proper understanding of the effect of printing parameters (e.g., extrusion temperature, build platform temperature, printing speed, and layer height) and physicochemical properties of polymeric filament is essential [14]. Indeed, several biocompatible polymers have been used to produce filaments, such as ethyl cellulose, polycaprolactone, polylactic acid polyvinylpyrrolidone, and polyvinyl alcohol (PVA) [15,16]. This last polymer is one of the most used due to its biodegradability and stability when extruded through the printing nozzle [14,15,16,17]. However, the loading of a drug in PVA filament constitutes a challenge due to the requirement of suitable physicochemical properties for extrusion and printing (e.g., crystallinity, melting point, glass transition temperature, etc.). Moreover, relatively low drug loading was achieved by the methods usually used (i.e., incorporation of the drug during the production of PVA filament by hot-melt extrusion and impregnation process) [18,19]. In this regard, FDM 3D printing of hollow systems (similar to HGC) for oral administration represents a highly attractive strategy [11]. Among the alternatives explored for printed hollow systems (i.e., capsular devices, CDs), Melocchi et al. [20] manufactured CDs for oral pulsatile release based on hydroxypropyl cellulose composed of a cover and a body to be filled with a drug and assembled after printing. Maroni et al. [21] designed a two-compartment CD with soluble, swellable/erodible, and enteric soluble polymers to obtain successive drug release pulses. The CDs were filled and assembled after the printing stage. Kempin et al. [22] produced a CD body in a gastro-resistant cellulose acetate phthalate and filled it with a pre-printed tablet containing a thermo- and acid-labile drug. Posteriorly, the FDM process continued to complete the top of the CD in a nearly insoluble polycaprolactone. Charoenying et al. [23] created a floating CD consisting of a cover and body in PVA followed by a thermal crosslinking process. Then, a commercial HGC containing amoxicillin was embedded inside the CD, achieving a gastro-retentive drug delivery system. Smith et al. [24] printed CDs in PVA to assess the regional absorption of a new drug in preclinical studies. In this case, the printing process was stopped to allow manual filling of the CD with the drug. Then, the process resumed to close the structure. Basa et al. [25] also printed PVA-CDs with and without orifices to evaluate the behavior of PVA under in vitro drug dissolution conditions. From these relevant contributions, it can be observed that the design and production of CDs by FDM 3D printing has not yet been explored as an alternative technology for magistral compounding. In previous work, we provided a design of a PVA-CD by FDM printed, which was filled (with sodium cromoglicate, an antiallergic drug) and closed in the same procedure [26]. To continue the development of FDM 3D printed PVA-CDs for the potential adoption in magistral compounding, the objective of this work was the design and production of immediate drug release PVA-CDs of similar dimensions to a commercial HGC (size n° 0) with different wall thicknesses (0.2, 0.3, 0.4, 0.6, and 0.9 mm). The designs were printed and filled with an antihypertensive model drug (losartan potassium) during the printing process. To this end, different printing parameters, such as extrusion temperature, printing speed, material flow rate, and nozzle diameter were investigated. Then, physical characterizations, disintegration time, and in vitro dissolution studies of the CDs were performed. The CD with the wall thickness that showed the disintegration time and dissolution profile most similar to the HGC was designed and printed in different sizes (similar to HGC sizes n° 1 and 2) and also used in compounding pharmacy. In addition, mathematical modeling and prediction of drug release has become an increasingly vital area in both academic and industrial sectors, holding immense future potential. The in silico optimization of new drug delivery systems presents the potential to significantly enhance accuracy and ease of application. In fact, mathematical predictions enable prior estimates of the required composition, geometry, dimensions, and preparation methods of the respective dosage forms. Thus, one of the major driving forces for the use of mathematical modeling in drug delivery is to save time and to reduce costs since the number of required experimental studies to develop a new and/or optimize an existing product can significantly be reduced [27]. Hence, in this contribution, a mathematical function derived from the Weibull model including a functionality with the CD wall thickness was proposed and adjusted to predict the drug release with the aim of aiding the design process. 

## 2. Materials and Methods

### 2.1. Materials

The production of the CDs involved the use of a 1.75 mm diameter PVA filament (e-SUN, Shenzhen, China). Losartan potassium (LP, 99.8% purity over anhydrous basis) was incorporated into the HGCs and CDs. HGC sizes n° 0, 1, and 2 (Parafarm, Saporiti, Buenos Aires, Argentina) were used to compare with the CDs’ physicochemical behavior. Distilled water was used as the dissolution medium.

### 2.2. Capsular Devices Design

**Figure 1:**
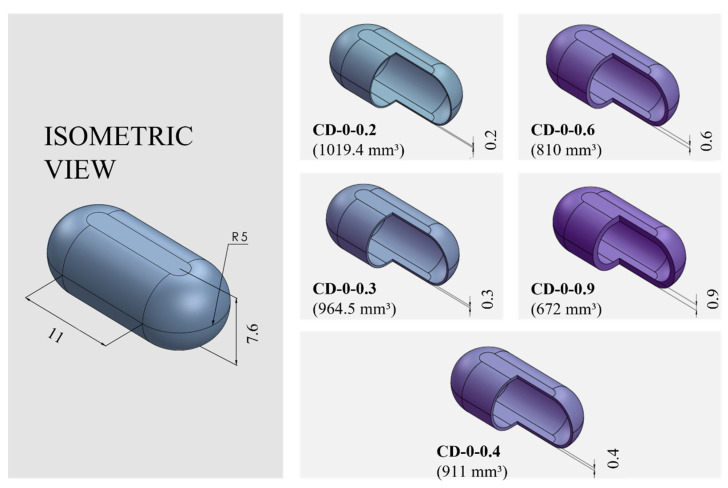
Capsule Device (CD) design n° 0 (CD-0) with different wall thicknesses (CD-0-0.2, CD-0-0.3, CD-0-0.4, CD-0-0.6, and CD-0-0.9) and their corresponding internal volumes (in mm^3^) and dimensions (in mm).

**Figure 2:**
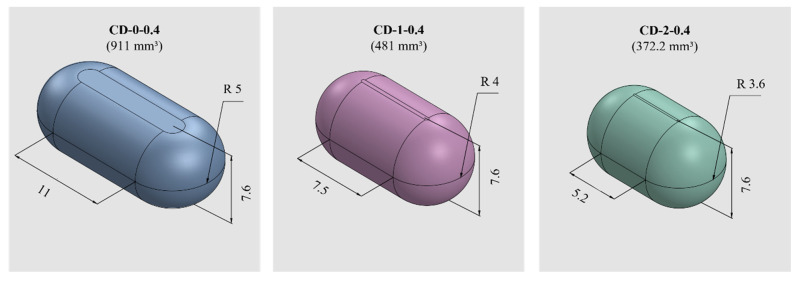
CDs of different sizes with the corresponding internal volumes (in mm^3^) and dimensions (in mm).

PVA-CDs of different wall thicknesses (*w*) and sizes were designed using the free CAD software OnShape (Boston, MA, USA) [28]. CDs with *w* of 0.2, 0.3, 0.4, 0.6, and 0.9 mm were designed to resemble an HGC of size n° 0. These were identified as HGC-0, CD-0-0.2, CD-0-0.3, CD-0-0.4, CD-0-0.6, and CD-0-0.9. Table 1 shows the internal volume of HCGs and CDs of different sizes. The CD-0-0.9 exactly followed the internal volume of HGC-0 (672 mm^3^); the other designs maintained the external dimensions of the CD-0-0.9 while slightly changing the internal cavity volume according to the variation in *w*. Figure 1 exhibits cut sections corresponding to all the designs n° 0 with different *w* (CD-0-0.2, CD-0-0.3, CD-0-0.4, CD-0-0.6, and CD-0-0.9). For the 0.4 mm *w*, two other sizes were designed following the internal volume of HGCs of size n° 1 and 2 (CD-1-0.4 and CD-2-0.4). In these designs, the height of CD-0-0.4 was maintained and the other dimensions were modified to mimic the HGCs’ internal volume (480 and 370 mm^3^ for HGC-1 and HGC-2, respectively). Figure 2 shows the isometric views of CD-0-0.4, CD-1-0.4, and CD-2-0.4. Both figures also show the internal volumes of each CD design. The resulting files were loaded into the Repetier Host V2.2.4 slicer program [29] and various printing settings were set accordingly.

### 2.3. Three-Dimensional Printing Process

The PVA-CDs were produced utilizing a Prusa type (Prusa I3 Hephestos, Buenos Aires, Argentina) FDM printer. The printing of the CDs consisted of three stages: first, the body was created, which included the first 64 layers of the piece. Then, the printing process was paused and 50 mg of losartan potassium powder was manually loaded. Once the CD was filled, the cover was printed by adding 11 further layers. All CDs presented equal height to maintain a constant number of 75 layers [26]. 

The printing process was modified for each design to obtain a completely sealed and flawless piece by changing variables such as extrusion temperature (*T_ext_*), printing speed (*V*), and material flow rate (expressed as percentage, *F*). It was also necessary to change the extrusion nozzle (i.e., nozzles with different diameters, *N_d_*) to be able to print thinner structures. Efforts were made to always use a nozzle with a diameter smaller than the *w* of the CDs. A print was successful when a full CD was obtained without failures. The printing efficiency (expressed as a percentage, *PE*) was determined based on the total number of successful prints divided by the total number of attempts [30]. A USB Digital Microscope M−DIG7 (Gadnic, Buenos Aires, Argentina), with magnifications ranging from 40× and 1000×, was used to verify that the CDs were satisfactorily sealed and flawless immediately after printing.

### 2.4. Physical Tests: Dimensions and Average Weight

To ensure the accuracy and reliability of the 3D printing process, an evaluation of the dimensions and weights of the CDs was carried out. To this end, at least 15 CDs were physically characterized by measuring their height and length using a digital caliper gauge (Hamilton Professional Brand, C30, Berrotarán, Argentina). The average values ± SD were reported for each parameter. 

The CDs filled with the drug were weighed individually with an analytical balance (Ohaus PA2202, Parsippany-Troy Hills, NJ, USA) to calculate the weight arithmetic average ± SD. Weight uniformity was assessed in accordance with the International Pharmacopoeia [31] for uncoated tablets, which stipulates that no more than two individual weights should deviate from the average by more than 5%.

### 2.5. Compression Test

A one-cycle compression test was performed using a Texture Analyzer (TA Plus Lloyd Instruments, Bognor Regis, UK) equipped with a 50 N load cell. The CDs and HGCs were compressed to 50% of their original height using a cylindrical probe (25 mm diameter) at a crosshead speed of 0.5 mm/s. After the set displacement, the probe returned to its original position at the same speed. The instrument measures the applied force and cell displacement at each instant of the test, providing accurate force–displacement data. Consequently, the compression work was defined as the area under the force–displacement curve. This work provides a quantitative measurement of the energy absorbed by the CDs during the compression test, which can be useful in evaluating its mechanical behavior. Measurements were taken in triplicate.

### 2.6. Disintegration Time

The study was carried out using a disintegration tester apparatus (Scout, Buenos Aires, Argentina) according to USP 44-2021 [32]. All the CD designs and the commercial HGCs were placed individually in each of the six tubes of the basket rack. Sinkers were used to prevent floating. The tests were performed with the beaker filled with distilled water (800 mL) at 37.0 ± 0.5 °C. The basket rack was raised and lowered into the water at a specified frequency (29 and 32 cycles per minute). The disintegration time (*DT*) was defined as the time at which the CDs and HCGs were visually broken. Results were expressed as mean *DT* (s) ± SD.

### 2.7. In Vitro Dissolution Study

The dissolution profile of the drug from the CDs of different *w* and sizes and the HCG sizes n° 0, 1, and 2 were evaluated using a Dissolution Apparatus II (708-DS, Agilent Technologies, Santa Clara, CA, USA) at a controlled temperature of 37 ± 0.5 °C, using 900 mL of distilled water as the dissolution medium. The rotational speed was set at 100 rpm [26]. To determine the dynamic release profile, samples were taken at the following predetermined times: 5, 10, 15, 20, 30, 45, 60, 75, 90, and 120 min. For each sample withdrawal, 5 mL were collected and, to ensure consistency, the withdrawn volume was replenished with an equivalent volume of fresh medium (Agilent 8000, Dissolution Sampling Station, Agilent Technologies, Santa Clara, CA, USA). Drug concentration was measured by a PDA UV/VIS Lambda 265 Spectrophotometer (PerkinElmer, Waltham, MA, USA) at 256 nm [32]. Experiments were performed in triplicate and the mean values are reported.

The similarity factor *f*_2_ was used to compare the dissolution profiles between the CDs with different *w* (Equation (1)), as follows:

(1) $$\;\;f_{2}=50\log\left\{100{\left[1+{\left(\frac{1}{n}\overset{n}{\underset{t=1}{\sum }}{\left(R_{t}-T_{t}\right)}^{2}\right)}^{2}\right]}^{-0.5}\right\}$$

where *n* is the number of dissolution time points and *R_t_* and *T_t_* are the percentages of drug released at each time point for the two dissolution profiles to be compared. An *f*_2_ value between 50 and 100 indicates a similarity between the two release profiles [33].

### 2.8. Drug Delivery Mathematical Modeling

A Weibull model (Equation (2)), frequently applied to the analysis of drug dissolution studies [34], was selected as the base model for fitting the experimental data. The model is expressed as

(2) $$C=100\left(1-e^{-\frac{t^{\beta }}{\alpha }}\right)$$

where *C* is the fraction (%) of drug released in time *t*, *α* is the scale parameter that defines the time scale of the process, *β* is the shape parameter that characterizes the curve as either exponential (*β* = 1), sigmoid, S-shaped, with upward curvature followed by a turning point (*β* > 1), or parabolic, with a higher initial slope and thereafter consistent with the exponential (*β* < 1) [35]. 

Recognizing the influence of capsule *w* on the drug release kinetics; the Weibull model was extended to include this variable, as it will be explained in detail in the results section (Section 3.2). This adds a new level of complexity to the model, allowing us to account for the effect of varying CD *w* on drug release.

The Weibull model was fitted to the experimental data, considering both time and *w*, using the method of least squares fitting. The general form of a multiple non-linear regression model is

(3) $$\;y=f\left(x_{1},x_{2},\ldots ,x_{n};\;Ɵ\right)+\epsilon$$

where *y* is the dependent variable; *x*_1_, *x*_2_, and *x_n_* are the independent variables; Ɵ represents the parameters of the model; and ϵ is the error term. Then, the optimization technique minimizes the sum of the squared differences between the model predictions and the actual dissolution data using the following expression [36,37]:

(4) $$min\sum_{i=1}^{m}{\left(y_{i}-f\left(x_{i1},x_{i2},\ldots ,x_{in};\;Ɵ\right)\right)}^{2}$$

where *m* is the number of observations in the dataset.

The fitting process was performed using MS Excel Office 365, obtaining optimal values for the Weibull parameters (*α* and *β*). The regression coefficient *R*^2^ was used to determine the goodness of fit. 

This comprehensive methodology not only explores the intricacies of the dissolution of CDs but also pioneers the integration of *w* as a critical variable for more accurate and personalized drug release predictions.

### 2.9. Statistical Analysis

One-way ANOVA at 95% (*p* < 0.05) was used to determine significant differences between the mean values of weight, compression test, and *DT*. In all the cases, it was previously verified that the population of samples had a normal distribution and met the criterion of homoscedasticity. In the case of the compression test, a pairwise comparison of the means was made using Tukey’s HSD test. 

## 3. Results and Discussion

### 3.1. Design: Wall Thickness and CD Size

#### 3.1.1. Three-Dimensional Printing Process

**Figure 3:**
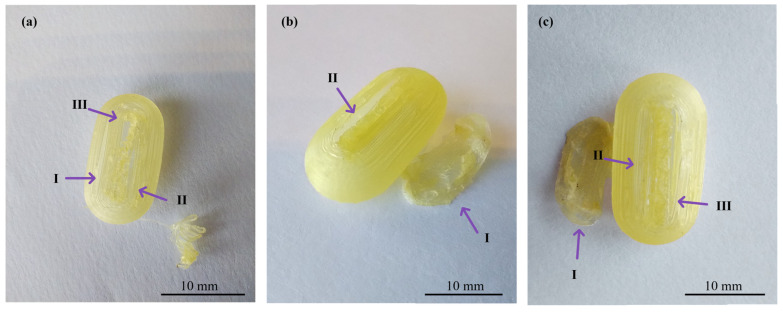
Images of three different CD-0-0.2-printed samples ((**a**), (**b**), and (**c**)) showing problems in adhesion to the printing platform (I), cracks between layers (II), and bridging (III).

**Figure 4:**
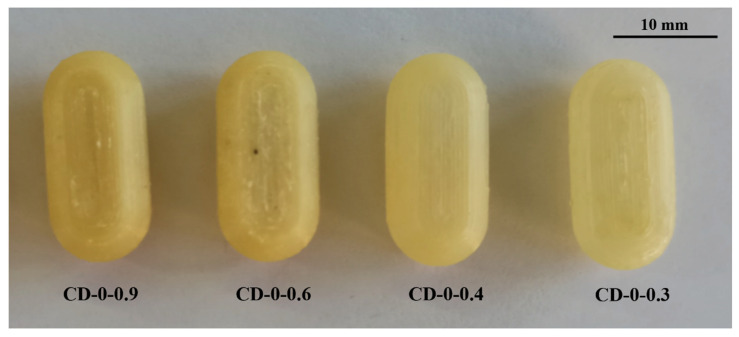
CD-0 with different wall thicknesses.

In the realm of personalized pharmacy, achieving precision and accuracy is a constant pursuit. For that reason, the manipulation of printing parameters becomes crucial in ensuring the production of flawless final products such as 3D printing CDs [38]. The CD printing process began with the printing of CD-0-0.9, inspired by the findings of our previous work [26]. CD-0-0.9 served as a solid base for printing thinner *w*. As shown in Table 2, for CD-0-0.9, it was necessary to increase *F* from the initial setting of 100% to 170% at layers 64 to 69 and to 150% at layers 70 to 75 to achieve a proper seal between the CD body and cover. *V* was also adjusted among the layers to achieve successful prints. Reducing this parameter allowed a smooth deposition of material on the layers, resulting in the complete closure of the CDs. Consequently, *V* was decreasing at layers 64 to 66 from 1200 to 250 mm/min, at layers 67 to 100 mm/min, and at layers 68 to 75 to 250 mm/min. This modification allowed optimum adhesion between the body and cover. This CD design (CD-0-0.9) achieved a *PE* of 100%. Nonetheless, as *w* decreased, it became more challenging to obtain a completely closed and flawless print. Hence, it was necessary to change some printing parameters. For all CD-0-0.6, CD-0-0.4, and CD-0-0.3 designs, it was necessary to modify *T_ext_* with respect to CD-0-0.9. This parameter was increased from 190 °C to 200 °C after manual drug filling (i.e., layers 64 to 75). For DC-0-0.6, despite *T_ext_*, no changes were made to the printing parameters compared to CD-0-0.9, achieving a *PE* of 100%. Although the CD-0-0.4 did not show any changes in *F* and *V* compared to CD-0-0.9, a change in the nozzle diameter (*N_d_* from 0.4 mm to 0.2 mm) was required to print a thinner *w* without imperfections. This design achieved a *PE* of 85%. Regarding CD-0-0.3, it was also printed using an *N_d_* of 0.2 mm. Although closed CD-0-0.3 was able to be obtained for physical and biopharmaceutical analysis, the *PE* was less than 20% (i.e., 80% of CD-0-0.3 failed during the printing process). Furthermore, various efforts were made to print the CD-0-0.2. Twenty-two different sets of printing parameters were tested, adjusting *F*, *V*, and *T_ext_* to obtain a successful print. Nonetheless, failures were observed in all the CDs. To counteract the problems of adhesion to the printing platform for the first layers, *F* was increased from 100 to 180%, resulting in a more robust base, albeit still presenting failures in the subsequent layers (Figure 3b(I),c(I)). For the final layers of the cover, even if different *F* settings were tested (between 170 and 300%), cracks and adhesion problems were unsolved (Figure 3a(II),b(II),c(II)). In some cases, the cover was not completely closed due to bridging failure, resulting in unwanted gaps (Figure 3a(III),c(III)). In additive manufacturing, bridging the printing of a cantilevered part of the 3D-printed object is a particularly challenging process [39]. It should be noted that although changes were made to address the above-mentioned failures in the critical areas, it was not possible to solve the problems consistently. Finally, based on the aforementioned results, design CD-0-0.4 was considered as the CD with the thinner *w* able to be printed. Figure 4 shows the printed CD-0 arranged in order of decreasing *w* (i.e., CD-0-0.9, CD-0-0.6, CD-0-0.4, and CD-0-0.3). Each CD is an identical replica of the next, emphasizing the precision of the printing process in reproducing the same dimensions.

**Figure 5:**
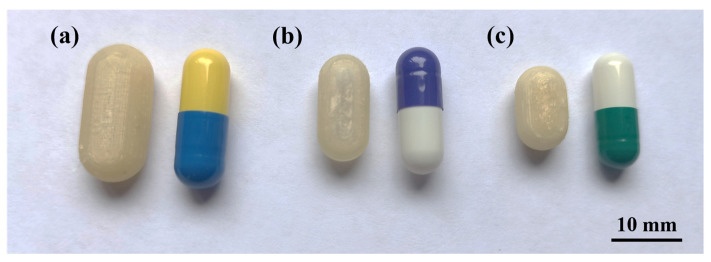
CD of different sizes compared with Hard Gelatin Capsules (HCG). (**a**) CD-0-0.4 and HGC n° 0, (**b**) CD-1-0.4 and HGC n° 1, and (**c**) CD-2-0.4 and HGC n° 2.

As mentioned in Section 2.2, two sizes were designed with the internal volume of HGCs of size n° 1 and 2 (CD-1-0.4 and CD-2-0.4). The same printing parameters used for CD-0-0.4 were appropriate to print CD-1-0.4 and CD-2-0.4, achieving a *PE* of 90% and 80%, respectively (Table 2). Figure 5 shows the printed CD-0-0.4, CD-1-0.4, and CD-2-0.4 compared to the corresponding HGC. Despite the dimensional variance, the CDs accurately replicate the internal volume of the corresponding HGC, as previously mentioned.

#### 3.1.2. Physical Tests: Dimensions, Weight, and Compression Performance

In terms of CD dimensions, the theoretical design dimensions and the printed devices were very similar for both length and height. The CDs with the largest errors were CD-0-0.3 and CD-1-0.4 but these errors were less than 3%. This small discrepancy indicates a high degree of accuracy in the printing process (Table 3).

The same trend was observed for the weights, showing no statistically significant differences between the mean weights of the CDs of equal *w* and size (*p* > 0.05). In addition, all the CDs fulfill the uniformity of weight pharmacopeia criteria for uncoated tablets (Table 3).

**Figure 6:**
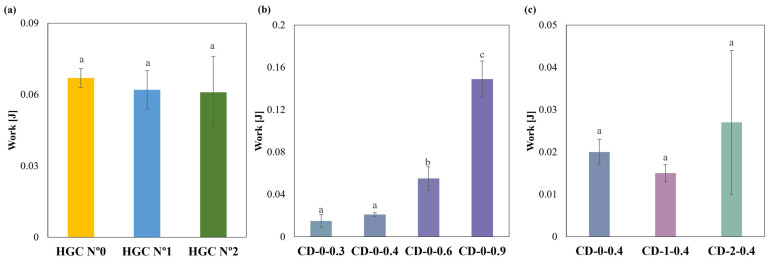
Compression work of (**a**) HGCs, (**b**) CDs of different *w*, and (**c**) CDs of different sizes (columns with the same letters indicate no significant difference among the compression work of the samples).

Regarding compression behavior, HGCs of sizes n° 0, 1, and 2 exhibited similar values of compression work. No statistically significant differences were observed (*p* > 0.05), indicating uniform mechanical properties regardless of size (Figure 6a). Notably, the compression work of the CDs with different *w* showed significant differences (*p* < 0.05) (Figure 6b). The Tukey´s HSD test exhibited statistical differences in CD-0-0.3 with CD-0-0.6 and CD-0-0.9; CD-0-0.4 with CD-0-0.6 and CD-0-0.9; and CD-0-0.6 with CD-0-0.9, showing no difference in CD-0-0.3 with CD-0-0.4 (Figure 6b). When comparing CD-0-0.4, CD-1-0.4, and CD-2-0.4, no significant differences in compression work (*p* > 0.05) were observed (Figure 6c). This suggests that *w* has a greater influence on compressive work response than size. The observed differences in compression work among the commercial HGCs and the printed CDs could be attributed to differences in the material and manufacturing processes. HGCs exhibit consistent compression behavior across different sizes due to uniform material properties and manufacturing standards. In contrast, the CD’s structural integrity is highly related to the printing settings and chosen design. Furthermore, the construction process layer-by-layer incorporates structure anisotropy that is reflected in lower compression works. On the other hand, the commercial HGCs’ higher compression work can be also attributed to the double layer of material presented in the compression area due to the body and cover assembly.

#### 3.1.3. Disintegration Time and In Vitro Dissolution Study

Table 4 shows the *DT* values of HGCs and CDs of different *w* and sizes. As can be seen, both HGCs and CDs exhibited *DT* values lower than 30 min (1800 s), meeting the requirement for immediate-release solid oral dosage forms [32]. Particularly, we observed that the *DT* of the CDs were longer than those of the HGCs (Table 4). The *DT* increased with *w*, which is expected since it increases the CD’s structural integrity. PVA is a water-soluble polymer that erodes into a physical hydrogel [25]. Indeed, the additional wall material may provide greater resistance to external forces due to a more viscous hydrogel formation, giving longer *DTs*. In relation to size, CD-0-0.4 showed the longest *DT* while *DT* for CD-2-0.4 was longer than for CD-1-0.4. In fact, *DT* for CD-2-0.4 and CD-1-0.4 present no significant differences (*p* > 0.05). This indicates that the effect of size is important for the biggest CDs, which are more difficult to erode (i.e., longer *DT*).

**Figure 7:**
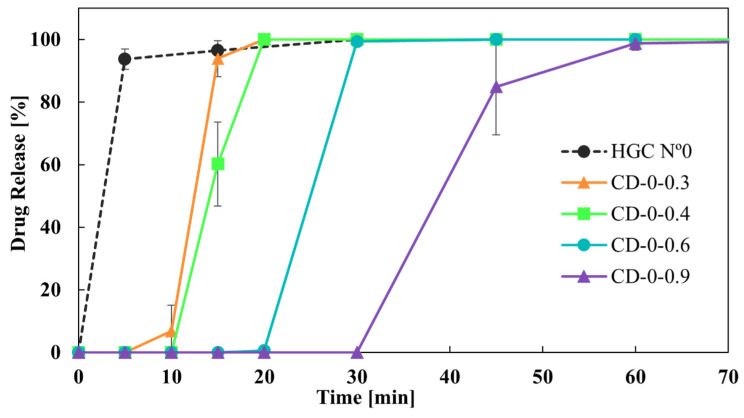
Drug release from CDs-0 of different wall thicknesses.

An in vitro dissolution study was conducted to evaluate the drug release from HGCs and CDs, which represent one of the most critical characteristics of dosage forms in formulation development. The similarity factor, *f*_2_, was chosen to compare the drug release profiles from the capsules due to its simplicity in assessing the global similarity of the dissolution curves [33]. Regarding the drug release from HGCs and CDs, Figure 7 shows the dissolution profiles of the CDs-0 with different *w* compared to HGC-0. It is observed that CD-0-0.3, CD-0-0.4, CD-0-0.6, and HCG-0 released more than 80% of the drug in 30 min, demonstrating it to be an immediate-release solid oral dosage form [40]. For CD-0-0.9, the *DT* was close to 30 min, suggesting it to be an immediate-release solid oral dosage form. However, regarding dissolution behavior, CD-0-0.9 presented a retarded drug release profile, with drug release starting at 30 min and approximately 80% of the drug being released at 45 min. This indicates that a higher *w* allowed a delayed drug release due to the presence of more PVA in the CD wall, which led to a hydrogel layer formation [25]. In addition, *f*_2_ was lower than 50 when comparing the release profiles between the CDs of different *w*, as well as between the CDs and the HCG-0 profile (Table 5). As can be seen, the variation in the *w* emerged as a significant factor affecting drug release.

As it was observed, both CD-0-0.3 and CD-0-0.4 drug release profiles were the closest to the HGC-0 one (Figure 7). However, it is important to note that CD-0-0.3 presented a lower *PE* than CD-0-0.4 (85% versus 20%). Thus, CD-0-0.4 was the design most similar to the HGC-0, feasible to be produced by 3D printing. 

**Figure 8:**
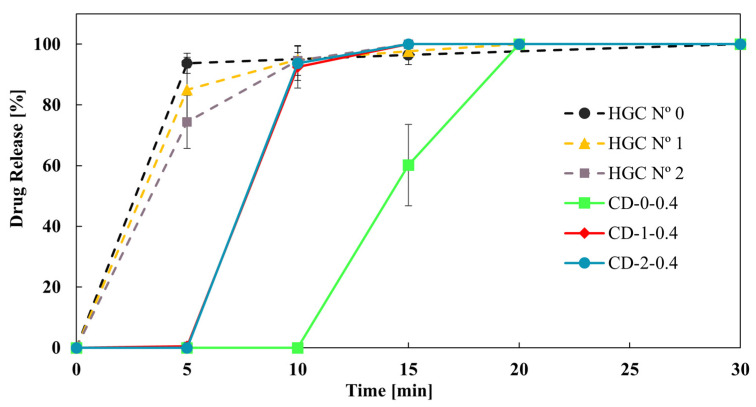
Drug release from CD-0.4 and HGCs of different sizes.

Finally, Figure 8 shows the effect of reducing the size of the CD-0-0.4 on the drug dissolution profiles. CD-1-0.4 and CD-2-0.4 showed more similar dissolution profiles to HGC than CD-0-0.4. This result was in concordance with the *DT* tendency (Table 4). In addition, the *f*_2_ value was higher than 50 between CD-1-0.4 and CD-2-0.4, showing similarity in the drug dissolution profile of these CDs. That is, a limit in drug release is achieved when reducing the size of the printed CDs, while all sizes correspond to immediate-release solid oral dosage forms [40]. Therefore, CD-2-0.4 could be a proper design to achieve an immediate drug release with less PVA consumption.

### 3.2. Drug Delivery Mathematical Modeling

As previously mentioned, a Weibull type dissolution model was applied to model drug release from CDs. In particular, the effect of *w* on the drug dissolution rate was investigated. To this end, the following exponential functions were proposed to correlate the *α* and *β* parameters of the Weibull model obtained for each dissolution profile with *w* (Equations (5) and (6)), as follows:

(5) $$\alpha =1.75E6w^{-0.56}$$

(6) $$\beta =3.69w^{-0.39}$$

**Figure 9:**
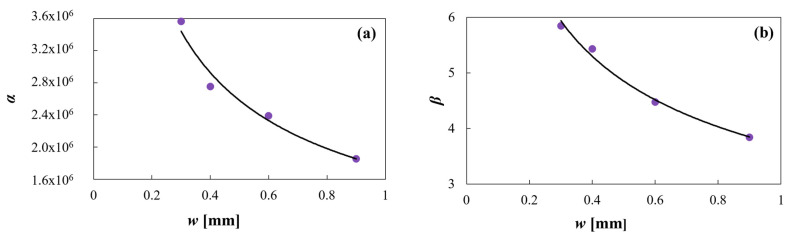
(**a**) Equation (5) model against *α* predicted data and (**b**) Equation (6) model against *β* predicted data.

As can be seen in Figure 9, the proposed exponential functions correctly fit the *α* and *β* data, being the *R*^2^ values 0.97 and 0.99, respectively. 

The final expression obtained of the modified equation of the Weibull model as a function of time and *w* is (Equation (7)), as follows:

(7) $$c=100\left(1-e^{-\frac{t^{3.69w^{-0.39}}}{1.75E6w^{-0.56}}}\right)$$

**Figure 10:**
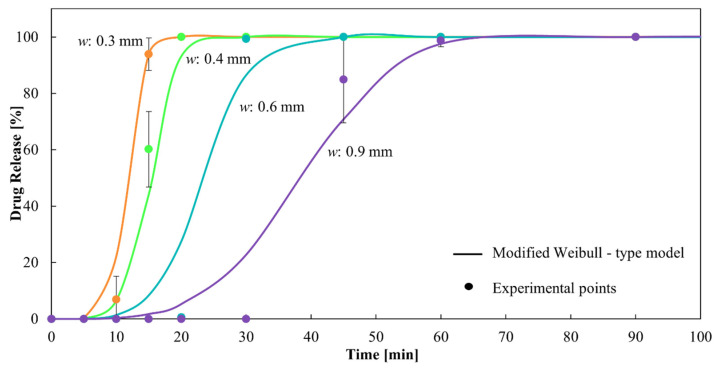
Dissolution profiles of CDs of different wall thicknesses from the modified Weibull-type model and experimental points.

It can be observed that *α* and *β* decrease with increasing *w*. The combined exponential decrease of both parameters, although with different orders of magnitude, allows the model to delay the drug release as *w* increases, in concordance with the experimental data. This can be observed in Figure 10, which present a dissolution profile predicted by the proposed model together with the corresponding experimental points. The corresponding *R*^2^ is 0.98, showing excellent fitting.

## 4. Conclusions

Capsular devices (CDs) with different wall thicknesses—*w*—(0.3, 0.4, 0.6, and 0.9 mm) and sizes (resembling hard gelatin capsules of size n° 0, 1, and 2) were designed and successfully 3D printed in polyvinyl alcohol by carefully optimizing the extrusion temperature, printing speed, material flow percent, and nozzle diameter. All printing parameters were critical for accomplishing a fully sealed body and covers, especially for the thinner wall thickness CDs. In fact, it was not possible to find a combination of printing settings that allowed us to successfully obtain a 0.2 mm CD, and a low percentage of printing efficiency was obtained for the 0.3 mm CDs. The physicochemical, pharmaceutical, and biopharmaceutical performance of the different CDs was evaluated with the aim of obtaining an immediate drug release profile (equivalent to HGCs) to be adopted in magistral compounding. As expected, the disintegration time (*DT*) of the CDs increased with increasing *w*. In concordance, slower drug release from the CDs was observed with increasing *w*. CD of 0.4 mm of *w* in the three sizes (CD-0-0.4, CD-1-0.4, and CD-2-0.4) showed proper uniformity of weight, short *DT*, an immediate drug-release, and a high printing efficiency emerging as promising devices to be used in future compounding pharmacy. In addition, a modified Weibull-type model is suggested, incorporating *w* as a new variable in the dissolution profiles predicted by the model. In this way, the proposed model facilitates the selection of a particular *w* based on the need to achieve a required dissolution rate in a specific time. As future work, studies regarding the stability of the CDs are required, as well as in vivo release studies. Furthermore, considering the importance of fully automatizing the printing process, the development of an automatic solid filling step, integrated into the CD production, is desirable for a promising application in compounding pharmacy.

## Figures and Tables

**Table 1:** The internal volume of Hard Gelatin Capsules (HGCs) and Capsule Devices (CDs) of different sizes.

	Internal Volume [mm^3^]		
	Size 0	Size 1	Size 2
HGC	670	480	370
CD-0-0.2	1019.4		
CD-0-0.3	964.5		
CD-0-0.4	911	481	372.2
CD-0-0.6	810		
CD-0-0.9	672		

**Table 2:** Printing parameters for CDs of different wall thicknesses and sizes. The numbers after each printing parameter indicate the layers affected by the change.

	*T_ext_* [°C]		*V* [mm/min]			*F* [%]		*N_d_* [mm]
Design	*T_ext_*1–63	*T_ext_*64–75	*V*64–66	*V*67	*V*68–75	*F*64–69	*F*70–75	
CD-0-0.3	190	200	250	100	250	170	150	0.2
CD-0-0.4	190	200	250	100	250	170	150	0.2
CD-0-0.6	190	200	250	100	250	170	150	0.4
CD-0-0.9	190	190	250	100	250	170	150	0.4
CD-0-0.4	190	200	250	100	250	170	150	0.2
CD-1-0.4	190	200	250	100	250	170	150	0.2
CD-2-0.4	190	200	250	100	250	170	150	0.2

**Table 3:** Dimensions and weights of CDs.

	CAD Design			Printed CD					
	CD-0	CD-1-0.4	CD-2-0.4	CD-0-0.9	CD-0-0.6	CD-0-0.4	CD-0-0.3	CD-1-0.4	CD-2-0.4
Weight [mg]				645.7 ± 8.7	451.7 ± 3.3	426.6 ± 4.4	346.9 ± 7.5	175.2 ± 6.7	145.5 ± 3.32
Height [mm]	7.6	7.6	7.6	7.7 ± 0.09	7.66 ± 0.07	7.61 ± 0.13	7.28 ± 0.10	7.79 ± 0.05	7.65 ± 0.24
Length [mm]	21	15.5	12.4	20.53 ± 0.01	20.61 ± 0.06	20.43 ± 0.14	20.68 ± 0.15	15.01 ± 0.02	12.38 ± 0.16
Error % ^a^				1.8	1.34	1.42	2.83	2.86	0.43

^a^ Average of the absolute errors $\frac{1}{2}\sum_{i=1}^{2}\left|X_{cad}-X_{i}\right|\times 100$. Error calculated between theoretical and experimental average of height and length. *X_cad_* is the dimension of the CAD design, *X_i_* is the mean value of each sample dimension, and *i* represents each measured variable.

**Table 4:** Disintegration times of CDs and HGCs.

HGC	*DT* [s]	CD	*DT* [s]
0	79 ± 14	0-0.9	1510 ± 54
		0-0.6	901 ± 71
		0-0.4	524 ± 25
		0-0.3	382 ± 44
1	73 ± 12	1-0.4	190 ± 45
2	66 ± 9	2-0.4	259 ± 19

**Table 5:** *f*_2_ values from the comparison of CD-0 of different wall thicknesses and HCG-0.

	*f*_2_				
	CD-0-0.3	CD-0-0.4	CD-0-0.6	CD-0-0.9	HGC N° 0
CD-0-0.3		48.1	18.2	13.4	19.3
CD-0-0.4			21.7	15.6	17.7
CD-0-0.6				24.9	10.5
CD-0-0.9					7.9

## Data Availability

Data are contained within the article.
